# Impact of a national HPV vaccination programme for preadolescent girls on cytology screening performance and CIN2+ incidence: five-year population-based cervical screening results from Slovenia

**DOI:** 10.1016/j.lanepe.2024.101203

**Published:** 2024-12-28

**Authors:** Abyan Irzaldy, Tine Jerman, Inge M.C.M. de Kok, Jan A.C. Hontelez, Harry J. de Koning, Erik E.L. Jansen, Urška Ivanuš

**Affiliations:** a Department of Public Health, Erasmus MC, University Medical Center Rotterdam, the Netherlands; b Department of Cervical Cancer Screening, Epidemiology and Cancer Registry, Institute of Oncology Ljubljana, Zaloška cesta 2, 1000, Ljubljana, Slovenia; c Heidelberg Institute of Global Health, Universitätsklinikum Heidelberg, Heidelberg, Germany

**Keywords:** Early detection of cancer, HPV, Vaccination, Vaccine, Screening, Mass screening

## Abstract

**Background:**

HPV vaccination is most efficacious in preventing cervical cancer and its precursors when administered during preadolescence. Because in Slovenia women are invited for cytology screening from age 20, women targeted for preadolescent HPV vaccination have been screening-eligible since 2018. We aim to assess the impact of preadolescent HPV vaccination programme on cytology screening performance and CIN2+ incidence.

**Methods:**

This is a cohort study using data from Slovenia's cervical screening registry. We compared the incidence of high-grade lesions (CIN2 or worse) between women in the first vaccine-targeted birth cohort (1998–2001) and in the latest non-vaccine-targeted birth cohort (1994–1997). We calculated the Incidence Rate Ratio (IRR) of CIN2+ using Poisson regression. To identify a possible shift in the harms-benefits ratio of screening, we compared the following screening performance indicators using chi-square tests: colposcopy referral rate (CRR), positive predictive value (PPV) of low-grade and high-grade results, and CIN2+ detection rate.

**Findings:**

The annual quadrivalent vaccine coverage in vaccine-targeted cohort was between 48.7% and 55.2%. The CIN2+ incidence was substantially lower in vaccine-targeted cohort (IRR 0.58 95% CI 0.49–0.69). Screening indicators were significantly lower for the vaccine-targeted cohort: direct CRR (0.47% [118/25,185] vs. 0.68% [206/30,181]), PPV (26.8% [30/112] vs. 39.0% [76/195]), and CIN2+ detection rate (0.31% [79/25,185] vs. 0.55% [165/30,181]) (p < 0.05).

**Interpretation:**

Even under imperfect vaccination coverage of around 50%, CIN2+ incidence in the vaccine-targeted cohort was observed to be 42% lower. Furthermore, the harms-benefits ratio of cervical screening deteriorates, marked by lower PPV and detection rate. This warrants an adaptation in screening algorithms in vaccinated cohorts.

**Funding:**

European Union Horizon 2020.

**Research in context**

**Evidence before this study**

We performed a literature search in Pubmed on June 2024 using the syntax (((Vaccination Program) OR (Vaccination Programme)) AND (HPV)) AND (Screening) for studies published between January 2018 and June 2024. We found several studies reported the impact of national/regional Human Papilloma Virus (HPV) vaccination programme on their cervical screening programme. Two studies focusing exclusively on the population-level impact of a national HPV vaccination programme targeting girls. Those two studies were conducted in countries with high coverage such as Denmark and Norway. The rest of the studies included women who were vaccinated at older ages, which is known to be less effective. No study investigated the impact of preadolescent HPV vaccination on both screening performance indicators and incidence of CIN2+ (Cervical Intraepithelial Neoplasia 2 or worse). Using the Slovenia's cervical screening registry, it is currently already possible to investigate the real-world impact of preadolescent HPV vaccination on screening outcomes, which include both the CIN2+ incidence and cytology screening indicators.

**Added value of this study**

To our knowledge this is the first study which exclusively evaluate the impact of a national HPV vaccination programme targeting preadolescent girls, in a country with lower HPV vaccination coverage (around 50%), on cervical screening outcomes which also include screening indicators and CIN2+ incidence. Offering HPV vaccination to preadolescent girls through organized and nationally implemented HPV vaccination programme, even with imperfect but substantial vaccination coverage, is associated with a substantially lower CIN2+ incidence. Secondly, cervical screening results of women which were previously targeted for preadolescent HPV vaccination showed lower positive predictive value for CIN2+. More primary cytology screening examinations also need to be performed to detect one case of CIN2+ (lower detection rate), which shows that cytology screening may be less efficient in vaccine-targeted population. These performance indicators demonstrate a less favourable harms-benefits ratio of cytology screening in a vaccine-targeted population.

**Implications of all the available evidence**

Previous studies have shown the substantial impact of scaling up HPV vaccination programme which may include catch-up vaccination programme and/or vaccination at older ages. This study has shown that even with imperfect coverage of HPV vaccination targeting preadolescent girls, substantially lower incidence of CIN2+ is already observed. Thus, vaccinating population through organized and publicly funded programme should be encouraged and efforts to increase vaccination coverage should be given. The deteriorating harms-benefits ratio of cytology screening observed in vaccine-targeted population found in this study may require further considerations on potential interventions such as a switch to HPV screening and/or risk-based screening.

## Introduction

Following the evidence on the efficacy and safety of Human Papilloma Virus (HPV) vaccination against cervical cancer and its precursors,^1^, ^2^, ^3^, ^4^ over 107 countries have implemented national HPV vaccination programmes since 2007.^5^^,^^6^ The primary target of HPV vaccination programmes is preadolescent girls, typically between 9 and 14 years old,^5^ as the efficacy of the vaccine is highest prior to sexual debut.^7^^,^^8^ These vaccination programmes will likely influence cervical cancer screening programmes which have been running several decades in many countries. However, the impact of preadolescent HPV vaccination programmes on cervical screening outcomes is largely unknown because many countries have not yet invited women, who were vaccinated as preadolescents, to screening. Majority of published real-life evidence on the impact of HPV vaccination includes women who were vaccinated at older ages including in catch-up campaigns which is known to be less effective.^9^^,^^10^

The cervical cancer screening and HPV vaccination programmes in Slovenia provide a unique opportunity to evaluate the impact of routine HPV vaccination on screening outcomes. Slovenia introduced quadrivalent HPV vaccination as part of their national routine immunisation schedule for girls aged 11–12 in 2009.^6^ Slovenia also operates a well-organised cervical screening programme for women aged 20–64 years since 2003. Invitations are given every three years, with cytology serving as the primary screening modality. Slovenia is currently achieving a participation rate of around 70% and is among the European countries with the lowest burden of cervical cancer.^11^ This early implementation of preadolescent HPV vaccination, combined with a lower age for screening initiation, allows for a timely assessment of the impact of preadolescent HPV vaccination on screening outcomes. Women in specific vaccine-targeted birth cohorts have already reached the screening-eligible age since 2018 in Slovenia.

In monitoring screening programmes, it is crucial to consider different screening indicators, each providing unique information on outcomes in different parts of the screening pathway.^12^ For example, calculation of positive predictive value (PPV) may give us insights on potential harms such as unnecessary referrals and false positives.^13^ The benefit of screening can be estimated by calculating detection rates, as it provides an indication of the number of prevented cancer cases or deaths. These indicators are essential for understanding the harms-benefits ratio of screening, a concept central to evaluating cancer screening programmes. The comprehensive screening registration in Slovenia enables such detailed evaluations, making it possible to assess changes in performance and efficiency, particularly in vaccinated populations. Therefore, this study aims to evaluate the early impact of national HPV vaccination programme targeting preadolescent girls on cytology screening performance and the incidence of high-grade lesions (Cervical Intraepithelial Neoplasia [CIN] 2 or worse).

## Methods

### Settings

#### Slovenian HPV vaccination programme

The Slovenian HPV vaccination programme was launched in 2009 and has been offered to girls aged 11–12 years old. This school-based vaccination programme is funded through mandatory health insurance. During the first few years, girls were offered three doses of quadrivalent HPV vaccines at 0, 2, and 6 months intervals,^14^ but this was changed to two-dose quadrivalent HPV vaccination in 2014. In 2016, the programme switched to the 9-valent vaccine, and boys have been included in the vaccination programme since 2021.^15^ In this analysis, all included vaccine-targeted birth cohorts were offered the girls-only policy with three doses of quadrivalent HPV vaccines. The vaccination coverage of the observed vaccine-targeted birth cohorts was stable ranging between 48.7% and 55.2% annually.^16^ There has been no major organized catch-up HPV vaccination campaign in Slovenia.

#### Slovenian cervical screening programme

Since 2003, the cervical screening programme in Slovenia has been offered to Slovenian residents aged 20–64 years.^17^ The programme offers cytology screening every three years; however, after attending their first screening, women are invited again one year later if their screening results are negative. If no abnormality is found in these first two screening rounds, they can continue with a three-year interval.

Detailed management guidelines for women who test positive on the cytology screening test are published on the website of the screening organisation.^18^ Direct referral to colposcopy is given to women with high-grade squamous intraepithelial lesion (HSIL) cytology findings or higher. Women aged 20 or older who are found to have Atypical Squamous Cells of Undetermined Significance (ASC-US) are called for a co-test after 6 months. Management for women with LSIL is age-dependent, but because all women in our study were aged below 35 years, they were referred to a repeat cytology test after six months.^18^ Adherence to colposcopy referral has always been nearly 100%.

### Study design, participants, and data source

This is a retrospective observational study including women aged 20 to 24 who belong to the first few vaccine-targeted birth cohorts (birth years 1998–2001) and the latest non-vaccine-targeted birth cohorts (birth years 1994–1997) and participated in the screening programme during the observation period. The choice of age range (20–24) is determined due to a) women are eligible for screening invitation starting from the age of 20 in Slovenia, and b) based on the latest available data up to 31 December 2023, all women in the oldest vaccine-targeted birth cohort (born in 1998) were observed throughout their entire 24th year of age. Women who had diagnoses of high-grade lesions (CIN 2+) before reaching the age of 20 were excluded.

**Fig. 1:**
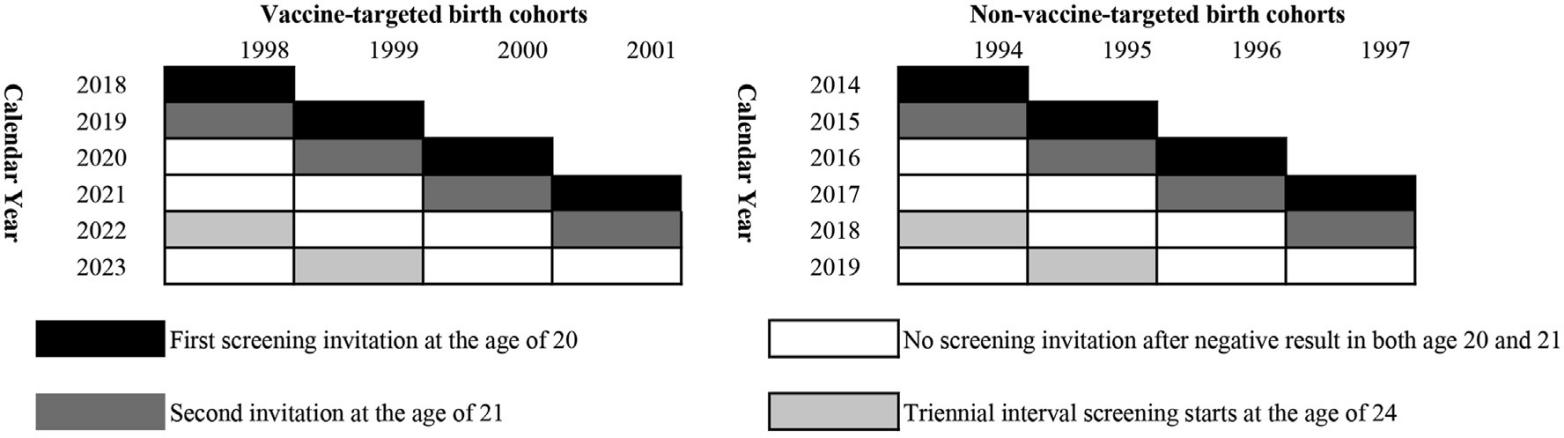
Diagram illustrating the observation period and a screening invitation timing scenario for women in vaccine-targeted birth cohorts and non-vaccine-targeted birth cohorts. The observation period ended on 31 December 2019 for women in non-vaccine-targeted birth cohorts and 31 December 2023 for women in the vaccine-targeted birth cohorts.

We assigned different observation periods for each vaccine-targeted and non-vaccine-targeted cohort. This is to ensure that both cohorts received the same number of screening invitations with the same timing and are in the same age range of 20–24 (Fig. 1). For women in the vaccine-targeted cohort, the observation period is 1 January 2018 to 31 December 2023. This is because the first vaccine-targeted birth cohort reached the age of 20 in the year 2018, and we used the registry data up to 31 December 2023. For optimal comparability, the observation period for the non-vaccine-targeted cohort was set to be between 1 January 2014 until 31 December 2019 (Fig. 1).

Individual-level data was obtained from the National Cervical Cancer Screening Programme and Registry (ZORA). ZORA registry contains all cytology, histology, and HPV test results of all women in Slovenia irrespective of the reason for the procedure or the source of payment. Reporting to ZORA registry is obligatory by law. Test results are updated on a nightly (three cytology laboratories), weekly, or monthly basis (two HPV, six cytology, and 11 pathology laboratories). Regular audit of data reported to the ZORA registry is routinely performed to check and verify any missing or illogical data. Furthermore, ZORA registry is connected with the central population registry and cancer registry.^19^

As this study relies only from cervical screening registry, information on vaccination coverage for each vaccine-targeted birth cohort was obtained from the national report of HPV vaccination coverage. The annual vaccination coverage among vaccine-targeted cohort was between 48.7% and 55.2%.^16^^,^^20^ Vaccination coverage refers to the percentage of individuals who have received all three doses of the HPV vaccine. Regarding examination coverage, the three-yearly examination coverage of women aged 20–24 from 2014 to 2023 remained stable, ranging from 81.1% to 86.4% (Supplementary Table S1). The reported examination coverage refers to the proportion of women in the target population who had at least one cytology test within the three-year screening interval.

### Outcomes and statistical analyses

#### Incidence of high-grade lesions (CIN 2+)

We define high-grade lesions as histologically confirmed CIN2+ (CIN2, CIN3, Adenocarcinoma in Situ (AIS), unspecified HSIL, and cervical cancer). All screening data were included from the day they attended their first cytology screening, until when they received any type of histologically confirmed CIN2+ diagnosis, a day before 25th birthday, deaths unrelated to cervical cancer, or the end of the group-specific observation period, whichever occurred first. Only deaths unrelated to cervical cancer are considered as competing events because cervical cancer is included in our definition of CIN2+. Thus, follow-up will stop at the time of a histologically confirmed CIN2+ diagnosis, including cervical cancer.

Crude incidence of high-grade lesions of women in vaccine-targeted and non-vaccine-targeted cohorts were calculated by dividing the number of CIN2+ cases by total person years during the observation period. Poisson regression was used to estimate the CIN2+ Incidence Rate Ratio (IRR) in vaccine-targeted compared to non-vaccine-targeted cohorts and its 95% confidence interval (CI). We visualized the cumulative incidence of CIN2+ among women in vaccine-targeted vs. non-vaccine-targeted cohorts over the follow-up period. To achieve this, we used Kaplan–Meier curve, treating the occurrence of CIN2+ diagnosis as the event of interest. Censoring was applied to account for competing events, such as death, reaching the age of 25, and the end of the observation period.

#### Screening performance indicators

For each cohort, we calculated the following five screening indicators for the first-time screens: direct colposcopy referral rate; indirect colposcopy referral rate; high-grade lesions positive predictive value for low and high-grade cytology results; and detection rate for high-grade lesions. We only analysed first-time screens because first-time screens have longer follow-up and the results of first-time screens are not affected by the number of previous screens. Thus, for women in vaccine-targeted cohort we excluded women who had less than one-year follow-up since their first-screen. The nominator and denominator for each calculated screening indicator can be seen in Table 1. Including only first-time screens and excluding women who had less than one year of follow-up since their first screen will consequently lead to fewer CIN2+ cases and fewer first-time screens compared to the numbers used in the incidence calculation.

**Table 1:** Definitions of nominators and denominators of calculated screening-performance indicators.

Indicators	Nominator	Denominator
Direct colposcopy referral rate	Number of women who were directly referred to colposcopy after the first-time screen.	Total number of first-time screens.
Indirect colposcopy referral rate	Number of women who were referred to colposcopy after repeat examination following the first-time screen.	Total number of first-time screens.
CIN2+ detection rate	All histologically confirmed diagnosis of high-grade lesions or worse (CIN2+) within one year following the first-time screen.	Total number of first-time screens.
CIN2+ PPV (low-grade cytology result)	All histologically confirmed diagnosis of high-grade lesions or worse (CIN2+) within one year following the first-time screen with low-grade cytology result.	Total number of first-time screens with low-grade cytology results.
CIN2+ PPV (high-grade cytology result)	All histologically confirmed diagnosis of high-grade lesions or worse (CIN2+) within one year following the first-time screen with high-grade cytology result.	Total number of first-time screens with high-grade cytology results.

For each indicator, we also calculated the confidence interval. *Chi-square tests* were performed to assess whether the differences in screening indicators between the vaccine-targeted and non-vaccine targeted cohorts are statistically significant (*p <* 0.05).

### Sensitivity analysis

In the non-vaccine-targeted birth cohorts, some amount of opportunistic vaccination, vaccination at older ages, and exposure to herd immunity is expected. To test the robustness of our analysis on the impact of preadolescent HPV vaccination programme on the incidence of high-grade lesions, we compared two older groups of non-vaccine-targeted birth cohorts, these are women who were born in 1990–1993 and 1986–1989. Secondly, due to minimum follow-up time needed in the calculation of screening performance indicators, women in vaccine-targeted cohort who do not have at least one year follow-up using data up to 31 December 2023 were excluded. To test the robustness of our calculation of screening performance indicators, we performed another sensitivity analysis which excluded also women in non-vaccine-targeted cohort who do not have at least one year follow-up using data up to 31 December 2019.

### Role of the funding source

This study is part of the EU-TOPIA-EAST project which was funded under the Global Alliance for Chronic Diseases (GACD) Cancer Research Programme by the EU-Framework Programme (Horizon 2020), project reference: 965014. The funder had no role in study design, data collection and analysis, decision to publish, or preparation of the manuscript.

## Results

There were 58,244 women born between 1994 and 2001 who participated in screening when they were between the age of 20 and 24 years. We excluded 36 women because they had a CIN2+ diagnosis before reaching the age of 20 years. Thus, we included in total 58,208 women with first-time cytology screening of which 30,181 belonged to the non-vaccine-targeted cohort and 28,027 to the vaccine-targeted cohort. There were fewer numbers of detected high-grade lesions in the vaccine-targeted birth cohort (n = 205) as compared to the non-vaccine-targeted birth cohort (n = 387) (Table 2).

**Table 2:** Characteristics of birth cohorts.

	Birth year	n	HPV vaccination coverage (%)^a^	Average age at first screening (in years)	Average follow-up time (in years)	Person-years	Detected CIN2+ cases (n)
Non-vaccine-targeted	1994	8513	–	21.4	3.6	30,715	147
	1995	8051	–	21.3	3.2	25,863	121
	1996	7250	–	21.1	2.4	17,264	66
	1997	6367	–	20.8	1.7	10,628	53
**Total**		**30,181**				**84,471**	**387**
Vaccine-targeted	1998	7858	48.7%	21.4	3.5	27,787	88
	1999	7340	55.2%	21.4	3.1	22,593	55
	2000	7010	54.9%	21.1	2.4	16,754	32
	2001	5819	48.9%	20.8	1.7	9702	30
**Total**		**28,027**				**76,835**	**205**

a The HPV vaccination coverage of organized programme targeting preadolescent girls. Based on official report.^20^

**Fig. 2:**
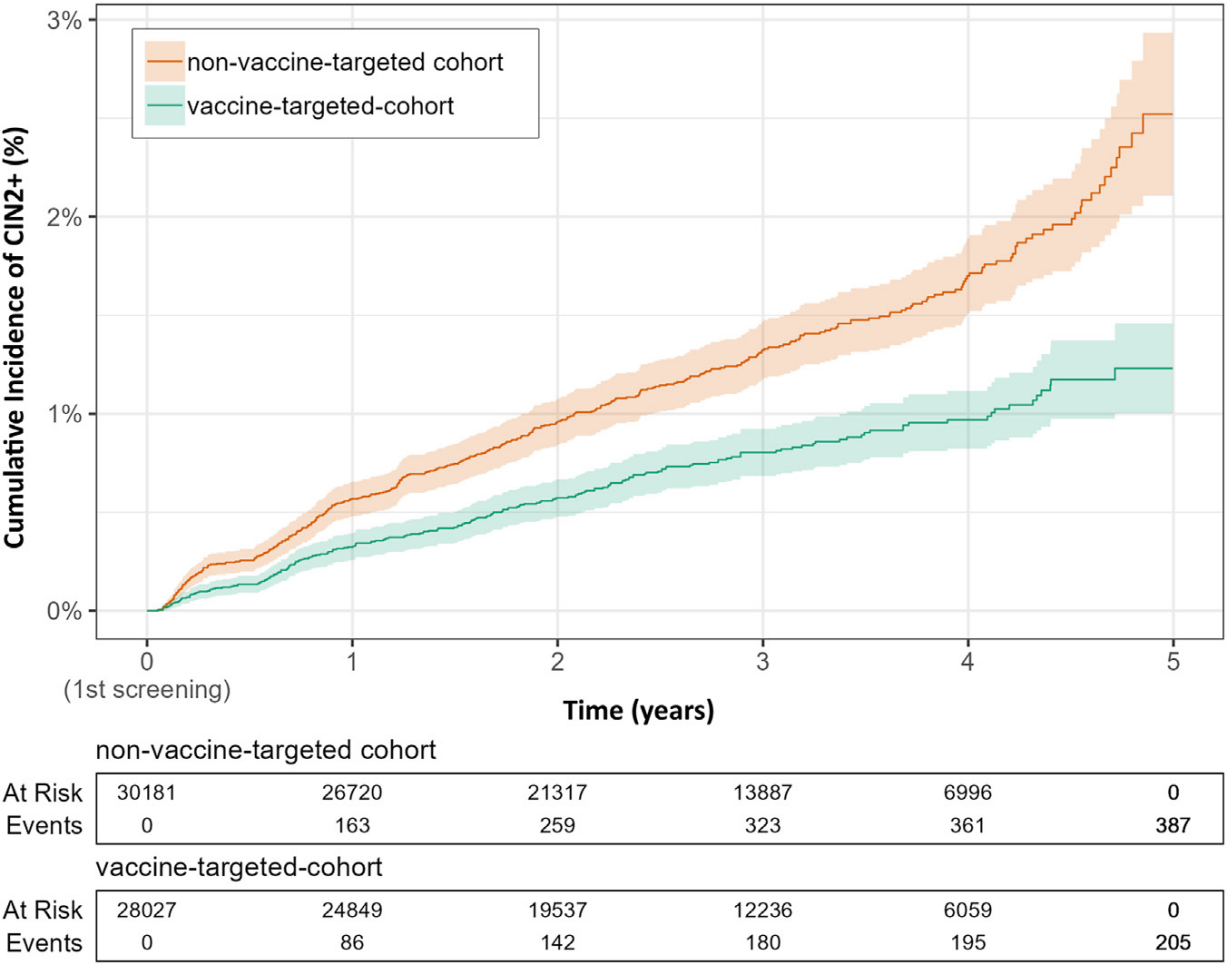
Kaplan–Meier curve: Cumulative incidence of high-grade lesions (CIN2+) between women in the vaccine-targeted cohort and non-vaccine-targeted cohort over the follow-up time.

We found a substantially lower incidence of high-grade lesions among women in the vaccine-targeted cohort (2.67 per 1000 person-years) as compared to women in the non-vaccine-targeted birth cohort (4.58 per 1000 person-years) (Table 3 and Fig. 2). The incidence rate of high-grade lesions in the vaccine-targeted cohort was 42% lower compared to that in non-vaccine-targeted cohort (IRR 0.58 95% CI 0.49–0.69). Further comparison of the studied non-vaccine-targeted cohorts (1994–1997) with the older non-vaccine-targeted cohorts (1986–1989 and 1990–1993) for the sensitivity analysis showed a largely similar incidence of high-grade lesions (Supplementary Figure S1).

**Table 3:** The Poisson regression result on the incidence of histologically confirmed high-grade lesions (i.e. CIN2+) among women in the non-vaccine-targeted and vaccine-targeted cohort.

	CIN2+ cases (n)	Person-years	Crude IR (per 1000 person years)	IRR (95% CI)	P-Value
Non-vaccine-targeted cohort	387	84,471	4.58	1.00 (Ref)	
Vaccine-targeted cohort	205	76,835	2.67	0.58 (0.49–0.69)	<0.001

IR, Incidence Rate of CIN2+ (per 1000 person years).

IRR, Incidence Rate Ratio of CIN2+.

CI, Confidence Interval.

**Fig. 3:**
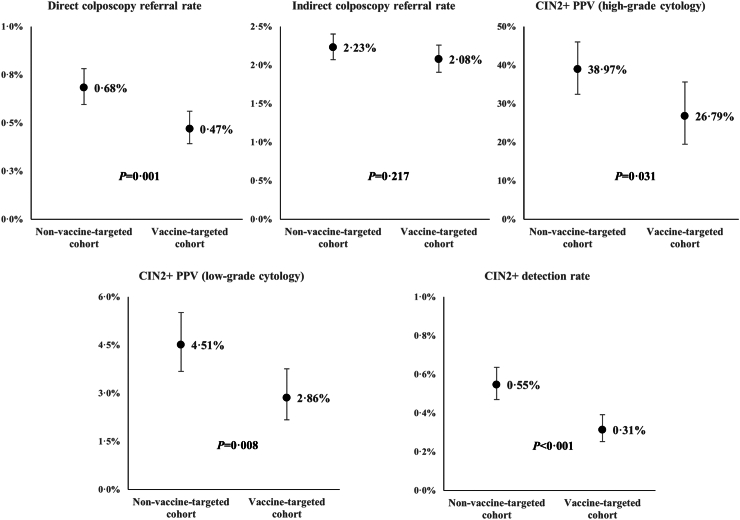
Cervical screening performance indicators in the non-vaccine-targeted and vaccine-targeted cohort, including 95% Confidence Interval (CI). *Note:* CIN2+ indicates cervical intraepithelial neoplasia 2 or worse; PPV indicates positive predictive value. The absolute number of the nominator and denominator for each calculated screening indicator are presented in Supplementary Table S4.

Our calculation of screening performance indicators showed that all indicators were lower in the vaccine-targeted cohort. Statistical significance was observed for all screening indicators (*p <* 0.05) except for indirect colposcopy referral rate (*p =* 0.217) (Fig. 3). Detection rate for CIN2+ in the vaccine-targeted cohort was lower (0.31%) than in the non-vaccine targeted cohort (0.55%) (*p* < 0.001). The CIN2+ positive predictive values for low-grade cytology results also demonstrated statistical significance (p = 0.008), while the CIN2+ PPV for high-grade cytology results also showed statistical significance (p = 0.031). The results of PPV and detection rate for CIN3+ showed similar findings to those of CIN2+ (Supplementary Table S2). The sensitivity analysis which exclude also women in non-vaccine-targeted cohort who had less than one-year follow up since their first screen using data up to 31 December 2019 showed very similar results (Supplementary Table S3).

## Discussion

We found a significant 42% lower incidence of histologically confirmed high-grade lesions (CIN2+) in a cohort of women of which around 50% was vaccinated at age 11 or 12, compared to a cohort of women who were not offered vaccination (IRR 0.58; 95% CI 0.49–0.69). We also found lower cervical screening performance indicator values such as direct colposcopy referral rate, CIN2+ positive predictive values for low and high-grade results, and CIN2+ detection rate in the vaccine-targeted cohort as compared to the non-vaccine-targeted cohort. This indicates more harms and less effective cytology screening, thus a less favourable harms-benefits ratio of cytology screening among women targeted for preadolescent HPV vaccination.

To our knowledge, this is among the first studies to look at population-level impact of HPV vaccination targeting preadolescent girls on CIN2+ detection and screening performance indicators. The identified reductions in incidence reflect both the direct effect of vaccination in vaccinated women and the indirect effect of herd immunity in unvaccinated women within the vaccine-targeted cohort. Our findings are in line with other studies which generally include women vaccinated at older ages.^9^^,^^10^^,^^21^ A large registry-based study from Sweden showed that the effectiveness of quadrivalent HPV vaccination against CIN2+ is 64% for women who were vaccinated before the age of 17.^3^ The observed effect in the Swedish study is larger than our study because the study comparing populations based on individual vaccination status. A Danish study reported lower effect of HPV vaccination with CIN2+ risk reduction of about 30%, which is possibly attributed mainly to the older vaccination age of 15.^22^ A recent study from Troms and Finnmark county in Norway, with preadolescent vaccination coverage ranging from 69.7% to 88.3%, showed that women in pre-vaccine cohorts had significantly higher CIN2+ risk (OR 9.2, 95% CI 5.9–13.8) than women in vaccinated cohorts.^23^ In the United Kingdom, a cohort of women who were vaccinated with bivalent HPV vaccination at age 12–13 with 88.7% coverage, showed 97% incidence reduction of CIN3.^9^ Our study adds to the current knowledge that, even with lower preadolescent HPV vaccination coverage of around 50%, a substantial reduction in the incidence of CIN2+ has already been observed.

The substantially lower incidence of CIN2+ in the vaccine-targeted cohort found in this study suggests the effectiveness of implementing preadolescent HPV vaccination despite the imperfect coverage. This finding highlights the importance of increasing vaccination coverage to further reduce the incidence of CIN2+ incidence. Thus, efforts to increase HPV vaccination coverage should be encouraged, especially in settings with currently imperfect vaccination coverage, to improve the population impact of vaccinating preadolescents.”

The decreasing incidence of high-grade lesions affects cytology screening performance and thus indicates a deteriorating harms-benefits ratio of cytology screening in women targeted for preadolescent HPV vaccination. This shift of the harms-benefits ratio of screening has been predicted by a previous modelling study.^24^ A registry study from Sweden which included screening outcomes of women vaccinated at older ages also showed a lower detection rate and PPV among vaccinated women.^25^ Our findings on colposcopy referral rates suggest a significantly lower direct colposcopy referral rate in the vaccine-targeted cohort, which implies a potential reduction in screening harms such as anxiety and discomforts associated with immediate colposcopies. However, the lower PPV in vaccine-targeted women indicates more unnecessary referrals among women targeted for preadolescent HPV vaccination. The reduced detection rate of CIN2+ suggests a decrease in the effectiveness of screening for high-grade lesions. This indicates reduced cytology screening benefits among women targeted for preadolescent HPV vaccination due to lower incidence of high-grade lesions in this population.

These shifts in harms-benefits ratio of cytology screening emphasize the need for adapted screening protocols for vaccinated women. A switch to HPV testing, possibly with genotyping triage, may help in better identifying high-risk HPV infections in the vaccinated population. This approach may also reduce unnecessary referrals.^26^^,^^27^ Furthermore, de-intensifying cervical screening among vaccinated women may decrease screening harms while still maintaining the efficiency of screening.^28^ For example, Italy has increased the age to start HPV testing from 25 to 30 for women vaccinated at the age of 12.^29^

There are several notable strengths of this study. First, the data came from routine screening practice in Slovenia. Thus, the data is objective by nature and therefore information bias could be minimized. Second, all women included in this study are affected by the same vaccination and screening policy during the observation period, therefore, no statistical adjustments for policy changes were needed. Furthermore, our approach to assessing population-level impact of HPV vaccination programme allows us to quantify the impact, taking into account imperfect coverage of vaccination. This will also be generalizable to other countries with similar circumstances.

One of the limitations of this study is that vaccinated women may be more likely to participate in screening than unvaccinated women,^30^^,^^31^ potentially leading to an overestimation of the impact of HPV vaccination programme, although women vaccinated opportunistically in non-vaccine-targeted cohort may also be more likely to participate to screening. Another limitation of this study lies in the absence of individual vaccination status information. The reported vaccination coverage in the vaccine-targeted cohort is the last-dose coverage. Therefore, we expect that there are actually women in vaccine-targeted cohort who receive protective effect of one or two doses of HPV vaccination.^32^ Furthermore, It cannot be ruled out that women may be vaccinated outside the routine vaccination programme. Thus, opportunistic vaccination activities in non-vaccine-targeted cohort may result in an underestimation of vaccination impact. However, in our sensitivity analysis, although we observed minor differences on CIN2+ incidence among non-vaccine-targeted birth cohorts (1986–1997), the differences in CIN2+ incidence between these cohorts were minimal and not significant, suggesting a minimal impact of opportunistic vaccination on the studied non-vaccine-targeted cohort of 1994–1997 (Supplementary Figure S1).

### Conclusions

In conclusion, we demonstrate that HPV vaccination programme targeting preadolescent girls, with around 50% coverage, is associated with 42% lower CIN2+ incidence. This signifies that, even with imperfect but substantial vaccination coverage, changes in CIN2+ incidence have been observed. Thus, efforts to increase vaccination coverage should be encouraged. Furthermore, the deteriorating harms-benefits ratio of cytology screening in women targeted for preadolescent HPV vaccination, marked by lower PPV and CIN2+ detection rate, indicates the necessity to revise screening policy for vaccinated cohorts.

## Contributors

**AI, TJ, EJ, UI:** Conceptualisation. **AI, TJ, EJ, UI, IdK, JH:** Methodology. **UI, TJ:** Data curation, Data Validation. **TJ:** Formal analysis and software. **AI, TJ:** Visualization. **AI:** Writing—Original Draft. **EJ, UI:** Supervision. **HdK**: Funding acquisition. **All Authors:** Investigation and interpretation, Writing—review and editing.

## Data sharing statement

Data on the examination coverage and other data from monitoring and evaluation of cervical cancer screening programme in Slovenia are freely available from ZORA programme website (https://zora.onko-i.si/en/monitoring-and-evaluation). Data on the burden of cervical cancer in Slovenia is freely available from the Slovenian Cancer Registry SLORA website (http://www.slora.si/en/). Anonymised individual data of this observational, population-based study will be available following the publication for at least 5 years together with investigators support, for investigators who will provide a methodological sound proposal, which was approved by the independent review committee, to achieve aims in the approved proposal. Proposals should be directed to Dr. Urška Ivanuš.

## Declaration of interests

All authors declare no competing interest.
