# Supplementary material for: Impact of a national HPV vaccination programme for preadolescent girls on cytology screening performance and CIN2+ incidence: five-year population-based cervical screening results from Slovenia
Source: Lancet Reg Health Eur. 2024 Dec 28;50:101203. doi: 10.1016/j.lanepe.2024.101203 (PMC11743916; doi:10.1016/j.lanepe.2024.101203)
Supplement: Supplementary Figure and Tables [file mmc1.docx]

**Supplementary Material**

**The impact of a national HPV vaccination programme for preadolescent girls on cytology screening performance and CIN2+ incidence: Five-year population-based cervical screening results from Slovenia.**

Abyan Irzaldy^1*^, Tine Jerman^2*^, Inge M.C.M. de Kok^1^, Jan A.C. Hontelez^1,3^, Harry J. de Koning^1^, Erik E.L. Jansen^1†^, Urska Ivanuš^2†^

1. Department of Public Health, Erasmus MC, University Medical Center Rotterdam, The Netherlands
2. Department of Cervical Cancer Screening, Epidemiology and Cancer Registry, Institute of Oncology Ljubljana, Zaloška cesta 2, 1000 Ljubljana, Slovenia.
3. Heidelberg Institute of Global Health, Universitätsklinikum Heidelberg, Heidelberg, Germany

*****† Authors contributed equally

**Table S1.** Three-yearly examination coverage among women aged 20-24 in the Slovenia’s cervical cancer screening programme.

**Table S2.** CIN3+ positive predictive values and detection rate between non-vaccine-targeted and vaccine-targeted cohorts.

**Table S3.** Sensitivity analysis excluding women in non-vaccine-targeted cohort who had less than one-year follow-up since their first screen.

**Table S4.** The absolute numbers of the nominator and denominator of calculated screening indicators based on the predefined definitions (Table 1).

**Figure S1.** Cumulative incidence of high-grade lesions (CIN 2+) of non-vaccine targeted cohort born between 1994-1997, non-vaccine-targeted cohort born between 1990-1993, non-vaccine-targeted cohort born between 1986-1989, and vaccine-targeted cohort 1998-2001 over the follow-up time.

**Table S1.** Three-yearly examination coverage among women aged 20-24 in the Slovenia’s cervical cancer screening programme.

| **Period** | **Women examined (N)** | **Women with permanent residence (N)** | **Examination coverage** |
| --- | --- | --- | --- |
| 2014-2017 | 42,889 | 49,409 | 86·8 % |
| 2015-2018 | 41,349 | 48,242 | 85·7 % |
| 2016-2019 | 40,415 | 47,354 | 85·3 % |
| 2017-2020 | 37,690 | 46,472 | 81·1 % |
| 2018-2021 | 38,411 | 45,866 | 83·7 % |
| 2019-2022 | 37,119 | 45,352 | 81·8 % |
| 2020-2023 | 37,165 | 44,975 | 82·6 % |

**Table S2.** CIN3+ positive predictive values and detection rate between non-vaccine-targeted and vaccine-targeted cohorts.

| **Indicators** | **Non-vaccine-targeted cohort** | **Vaccine-targeted cohort** | ***P*-value** |
| --- | --- | --- | --- |
| CIN3+ PPV (Low-grade cytology) | 2·1% (95% CI 1·5% - 2·8%) | 1·7% (95% CI 1·2% - 2·4%) | 0·391 |
| CIN3+ PPV (High-grade cytology) | 32·3% (95% CI 26·1% - 39·2%) | 19·6% (95% CI 13·3% - 28·0%) | 0·017 |
| CIN3+ detection rate | 0·34% (95% CI 0·28% - 0·42 %) | 0·20% (95% CI 0·15% - 0·27%) | 0·002 |

**Table S3.** Sensitivity analysis excluding women in non-vaccine-targeted cohort who had less than one-year follow-up since their first screen.

| **Indicators** | **Non-vaccine-targeted cohort** | **Vaccine-targeted cohort** | ***P*-value** |
| --- | --- | --- | --- |
| Direct colposcopy referral rate | 0·67% (95% CI 0·58% - 0·77%) | 0·47% (95% CI 0·39% - 0·56%) | 0·003 |
| Indirect colposcopy referral rate | 2·20% (95% CI 2·03% - 2·38%) | 2·08% (95% CI 1·91% - 2·26%) | 0·348 |
| CIN2+ PPV (Low-grade cytology) | 4·3% (95% CI 3·5% - 5·4%) | 2·86% (95% CI 2·2% - 3·8%) | 0·020 |
| CIN2+ PPV (High-grade cytology) | 39·0% (95% CI 32·0% - 46·4%) | 26·79% (95% CI 19·5% - 35·7%) | 0·035 |
| CIN2+ detection rate | 0·52% (95% CI 0·45% - 0·62 %) | 0·31% (95% CI 0·15% - 0·27%) | <0·001 |

**Table S4.** The absolute numbers of the nominator and denominator of calculated screening indicators based on the predefined definitions (Table 1).

| **Indicators** | | **Cohort** | **Nominator** | **Denominator** |
| --- | --- | --- | --- | --- |
| Direct colposcopy referral rate | | Vaccine-targeted | 118 | 25,185 |
|  |  | Non-Vaccine-targeted | 206 | 30,181 |
| Indirect colposcopy referral rate | | Vaccine-targeted | 523 | 25,185 |
|  |  | Non-Vaccine-targeted | 673 | 30,181 |
| CIN2+ detection rate | | Vaccine-targeted | 79 | 25,185 |
|  |  | Non-Vaccine-targeted | 165 | 30,181 |
| CIN2+ PPV (low-grade cytology result) | | Vaccine-targeted | 49 | 1,716 |
|  |  | Non-Vaccine-targeted | 89 | 1,975 |
| CIN2+ PPV (high-grade cytology result) | | Vaccine-targeted | 30 | 112 |
|  |  | Non-Vaccine-targeted | 76 | 195 |
|  |  | | | |


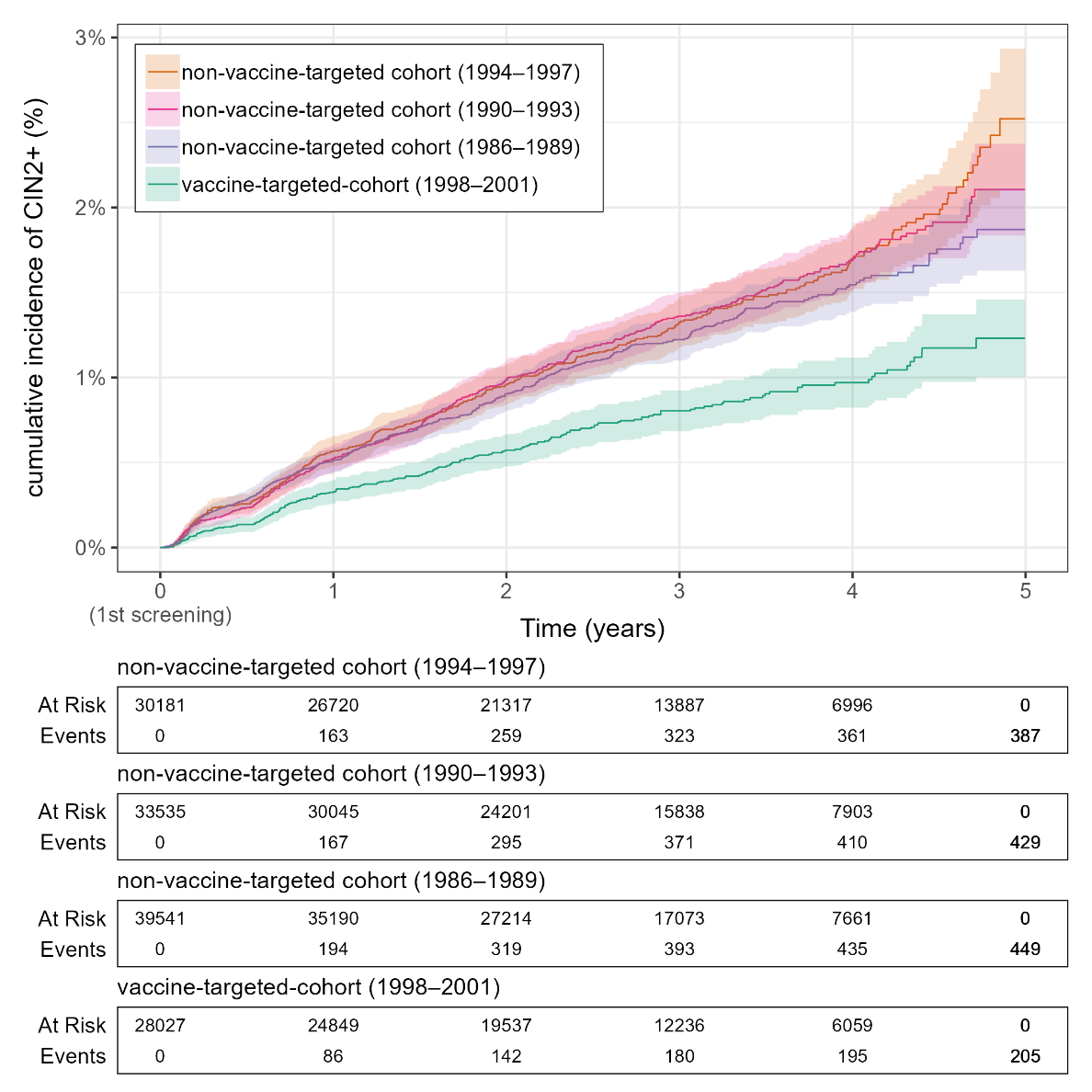


**Figure S1.** Cumulative incidence of high-grade lesions (CIN 2+) of non-vaccine targeted cohort born between 1994-1997, non-vaccine-targeted cohort born between 1990-1993, non-vaccine-targeted cohort born between 1986-1989, and vaccine-targeted cohort 1998-2001 over the follow-up time.
